# MULTIPLE SCLEROSIS AND SPASTICITY: THE ROLE OF ANAESTHETIC NERVE BLOCKS ON RECTUS FEMORIS MUSCLE. WHEN SHOULD STIFF KNEE BE TREATED WITH BOTULINUM TOXIN?

**DOI:** 10.2340/jrm.v56.40437

**Published:** 2024-08-05

**Authors:** Alessio BARICICH, Marco BATTAGLIA, Margherita B. BORG, Alberto LORO, Paola MORLINO, Lucia COSENZA, Michele BERTONI, Alessandro PICELLI, Andrea SANTAMATO, Thierry DELTOMBE

**Affiliations:** 1 Physical and Rehabilitation Medicine, Department of Health Sciences, Università del Piemonte Orientale, Novara, Italy; 2 Physical and Rehabilitation Medicine, “Ospedale Maggiore della Carità” University Hospital, Novara, Italy; 3 Physical Medicine and Rehabilitation, ASST Settelaghi, Varese, Italy; 4 Neuromotor and Cognitive Rehabilitation Research Center, Department of Neurosciences, Biomedicine and Movement Sciences University of Verona, Verona, Italy; 5 Spasticity and Movement Disorders “ReSTaRt” Unit, Physical Medicine and Rehabilitation Section, Policlinico Riuniti Hospital University of Foggia, Foggia, Italy; 6 Department of Physical Medicine and Rehabilitation, Université de Louvain, Centre Hospitalier Universitaire de Namur, Yvoir, Belgium

**Keywords:** botulinum toxin, multiple sclerosis, nerve blocks, personalized medicine, spasticity, rehabilitation

## Abstract

**Objective:**

To compare the effect of rectus femoris diagnostic motor nerve blocks (DNB) with anaesthetics and rectus femoris muscle botulinum toxin (BoNT-A) injection in multiple sclerosis patients with unilateral stiff-knee gait.

**Design:**

Prospective observational study

**Subjects/Patients:**

Multiple sclerosis patients in stable condition.

**Methods:**

Patients underwent evaluation before and 1 hour after the anaesthetic block, and 1 month after the botulinum injection. Assessment included a 10-m walking test, a 6-minute walking test, a timed-up-and-go (TUG) test, and a Baseline Expanded Disability Status Scale (EDSS). Post-DNB and post-BoNT-A satisfaction was measured with the global assessment of efficacy scale.

**Results:**

Fourteen patients with unilateral stiff-knee gait due to multiple sclerosis underwent a DNB, among whom 13 received botulinum injections in the rectus femoris muscle after a satisfying test result. Positive post-DNB results correlated with significant functional improvements after BoNT-A. Higher EDSS and longer time from diagnosis correlated with poorer post-DNB and post-BoNT-A absolute outcomes.

**Conclusion:**

DNB showed predictive value for BoNT-A outcomes, especially in the case of worse functional status. It effectively predicted endurance and walking speed improvement, while TUG showed greater improvement after botulinum. In cases of uncertain therapeutic benefit, nerve blocks may provide a valuable diagnostic support, particularly in patients with lower functional status.

## LAY ABSTRACT

Multiple sclerosis patients frequently suffer from lower limb spasticity and impaired gait, often associated with severe weakness. A typical finding is the “stiff-knee” gait pattern, due to rectus femoris muscle spasticity. Botulinum toxin is the treatment of choice to reduce spastic hypertonia but may also provide a further reduction in muscle strength with a pharmacological effect lasting up to 6 months. In this study, we assessed the efficacy of anaesthetic nerve blocks in predicting the possible expected effect of botulinum treatment on a shorter period of time (a few hours). Our results showed an interesting correlation between post-block gait functional tests and post-botulinum values, particularly in more compromised patients, suggesting a potential adjuvant role of nerve blocks in guiding the clinical decision.

Multiple sclerosis (MS) is a persistent and predominantly autoimmune condition affecting the central nervous system, characterized by inflammation, demyelination, and axonal loss that can occur even in the early stages of the disease. The progression and the clinical manifestation in MS vary significantly among individuals, and are a frequent cause of neurological disability among young people (1). The mean prevalence of MS worldwide is 35.9 per 100,000 people. Wealthy countries see the highest number of cases with a prevalence above 117/100,000 in America and 142/100,000 in Europe. Interestingly, from 2013 to 2020, these numbers underwent a significant increase of 54% and 34% respectively, underlining the clinical and social relevance of this condition (2).

A typical clinical manifestation of MS, involving almost every patient, is muscle weakness and chronic fatigue, negatively affecting patients’ autonomy and quality of life (1). Furthermore, MS is one of the leading causes of upper motor neuron syndrome, with approximately 80% of patients developing spasticity of variable severity, mainly affecting the lower limbs (3, 4). These 2 clinical manifestations (weakness and spasticity) can severely affect all the International Classification of Functioning, Disability and Health (ICF) domains.

For focal spasticity, the treatment of choice is botulinum toxin type A (BoNT-A), which provides a pharmacological denervation at the level of motor endplate lasting up to 4–6 months. Remarkably, the scientific literature concerning spasticity treatment is mainly focused on post-stroke spasticity (PSS) (5) so that evidence in MS patients relies on fewer studies. Nevertheless, BoNT-A has also proved to be effective in reducing spasticity, spasticity-related pain, and in improving quality of life (3, 6) in MS patients (7). However, some authors have raised concerns about possible differences in clinical and rheological features of spastic muscles between stroke survivors and MS subjects, questioning how spasticity may present significantly different clinical characteristics (8). Even though based on a specific muscle group (triceps surae), these findings suggest the need to embrace a particular approach in MS spasticity, somehow different from PSS, in terms of BoNT-A doses and treatment indication.

Another key element in MS is the relevance of chronic muscle weakness in terms of the disability condition, in particular gait impairment. Once again, researchers have mainly focused on plantar flexor (PF) muscles, which are frequently involved in cases of spasticity. In fact, reduced PF strength has been shown to be a better predictor of altered walking capacity than PF spasticity (9), establishing a crucial aspect to consider in MS functional assessment. Most importantly, these considerations could be extended to other body segments to improve clinical practice.

Considering the aforementioned BoNT-A mechanism of action concerning chemodenervation, additional weakening in an already weak muscle may occur alongside spasticity reduction. Therefore, some concerns may be raised regarding the possible negative influence of this treatment on muscle strength and consequently on functional performance (10, 11).

On this basis, it is essential to determine the benefit–risk balance before proceeding with long-lasting interventions. Before considering BoNT-A treatment, it is recommended to perform diagnostic anaesthetic blocks on the motor nerve branches supplying specific muscles (7). Technical procedures have been thoroughly described (12–14). In particular, in stroke survivors affected by spastic hemiparesis and stiff-knee gait pattern, diagnostic nerve blocks with anaesthetics (DNB) on rectus femoris (RF) motor nerve branches showed a significant correlation with subsequent BoNT-A treatment effects in terms of peak knee flexion and knee angular velocity (15). Therefore, RF nerve blocks may allow to predict the therapeutic outcome and the possible adverse effects of BoNT-A in a limited time frame of few hours. Collaterally, it is interesting to note that in MS, muscle tone alterations often present both a phasic (spasticity according to the original Lance definition [16]) and a tonic (spastic dystonia) pattern, which are variably prevalent and often coexistent in these patients (17). The correct identification of the spasticity phenotype may allow the possible treatment options to be better addressed.

The aim of this study was to compare, in patients with MS affected by RF spasticity and unilateral stiff-knee gait pattern, the effect of rectus femoris motor DNB and rectus femoris muscle BoNT-A injection in order to determine the predictive value of the of DNB before BoNT-A injection.

## METHODS

We conducted a prospective observational study. Inclusion criteria were diagnosis of MS with clinical and neuroradiological confirmation, age greater than 18 years, presence of lower limb spasticity graded 1+ or more on the modified Ashworth scale (MAS), unilateral stiff-knee gait, clinical indication for BoNT-A treatment, stable clinical and functional conditions at least 4 months earlier than and throughout the study time. Exclusion criteria were an inability to walk, Expanded Disability Status Scale (EDSS) ≥ 7, presence of comorbidities influencing gait capability, and test results. Patients were evaluated before the DNB (T0) and one hour after (T1). In case of subsequent BoNT-A treatment, experimenters conducted a reassessment 1 month after injection (T2) at the peak of the pharmacological effect. Each evaluation included a 10-m walking test (10mWT), a 6-min walking test (6MWT), a timed-up-and-go test (TUG), and the Expanded Disability Status Scale (EDSS) score (18). At T1 and T2, patient and/or caregiver satisfaction and operator satisfaction were assessed using the global assessment of efficacy scale (GAE) (19). The DNB targeting the afferent nerve branches of RF was performed using 2% lidocaine (1.5–2 mL) injected under ultrasonography and electrical stimulation guidance (14). We administered BoNT-A treatment no later than 2 weeks after a positive anaesthetic block outcome. Post-injection treatment remained unchanged from standard clinical practice including 10 sessions of rehabilitation treatment in a day-hospital setting. This study was approved by the local Ethics Committee with the register number 162/18, as a subgroup of unpublished data that formed part of a previously released study (3).

We used descriptive statistics to summarize the characteristics of the population. In particular, we reported categorical variables as absolute and percentage frequencies, and numerical variables as mean or median, if not normally distributed. The mean values of pre- and post-treatment 10mWT, 6MWT, and TUG were calculated to evaluate significant variations. The correlation coefficient was calculated to assess DNB predictability on BoNT-A effect and a paired *t*-test was performed to assess post-block and post-BoNT-A outcome overlap. We assessed the clinical significance of functional variables changes through specific minimal clinically important difference (MCID) and minimal detectable change (MDC) values, and the Pearson correlation coefficient (*r*) was calculated to assess anaesthetic block predictability. Specifically, for 6MWT the MCID considered was 34.4 m (20); for 10mWT the MCID was 0.16 m/s (21) and for TUG the MDC was 2.9 s (22). In the case of 6MWT and 10mWT we choose the MCID as current literature provides a validated threshold for chronic neurological disabilities, in particular for chronic stroke. Differently, for TUG, current evidence concerning MCID calculation relies on spine surgery patients (23, 24). Therefore, we opted to implement the MDC calculated on stroke survivors as the paradigm of neurological clinical condition. Our choice of an MDC for TUG was in coherence with the reference population used for 6MWT and 10mWT. Patients were finally stratified according to their EDSS score, based on the level of independence in walking (EDSS > 3.5 or ≤ 3.5).

## RESULTS

Fourteen patients completed the study. Thirteen showed post-DNB significant improvement on all the outcome variables and reported positive feedback on GAE by the patient/caregiver and the operator. These 13 patients were treated with BoNT-A, injected into the RF muscle. One patient did not receive the BoNT-A injection due to gait parameter worsening and GAE negative feedback after DNB. See Table I for demographic data.

**Table I:** Demographic characteristics of the study population

Variable	*n* = 14
Sex	
Males	2
Females	12
Positive DNB results with subsequent BoNT-A treatment	13
Negative DNB results	1
Age (years), mean (SD)	50.7 (9.7)
Time from diagnosis, mean (SD)	17.9 (11.0)
EDSS, mean (SD)	4.4 (1.3)

SD: standard deviation; EDSS: Expanded Disability Status Scale; DNB: diagnostic nerve block; BoNT-A: botulinum neurotoxin type-A.

A significant degree of overlap was observed between the improvement at T1 and at T2 for 6MWT and 10mWT. For TUG, changes at T2 were significantly higher than at T1 (Table II).

**Table II:** Global assessment of efficacy (GAE) scale and gait parameter variations between baseline and diagnostic nerve block (DNB) and between baseline and botulinum neurotoxin type-A (BoNT-A)

Factor	∆ after DNB		∆ after BoNT-A	
	Mean (SD)	MCID/MDC (*n*)	Mean (SD)	MCID/MDC (*n*)
10mWT (m/s), mean (SD)	0.07 (0.30)	4	0.12 (0.27)	5
6MWT (m), mean (SD)	30.23 (53.41)	6	66.00 (97.15)	6
TUG (s), mean (SD)	-1.37 (3.93)	1	-2.58 (4.44)*	2
GAE, mean (SD)	1.93 (0.92)	n/a	1.38 (0.51)	n/a

DNB can predict positive BoNT-A outcomes and underestimates post-BoNT-A TUG improvement.

10mWT: 10-m walking test; 6MWT: 6-min walking test; TUG: timed-up-and-go test; ∆: mean change from basal of each outcome variable; MCID: minimal clinically important difference; MDC: minimal detectable change; n/a: not applicable;

* statistical significance.

Higher EDSS scores and longer time since MS diagnosis correlated with worse functional outcomes post-block in all variables. This correlation was present with TUG only when considering post-BoNT-A evaluations (Table III).

**Table III:** Correlation coefficients between Expanded Disability Status Scale (EDSS) score and the time from diagnosis with outcome variables variations after diagnostic nerve block (DNB) and after botulinum neurotoxin type-A (BoNT-A)

Factor	EDSS		Time from MS diagnosis	
	After DNB	After BoNT-A	After DNB	After BoNT-A
∆ 10mWT	-0.33 *	-0.24	-0.42 *	-0.18
∆ 6MWT	-0.32 *	-0.18	-0.34 *	-0.02
∆ TUG	0.41 *	0.49 *	0.48 *	0.47 *

Higher EDSS and longer time from diagnosis negatively influence all post-DNB results, while affecting only TUG after BoNT-A.

10mWT: 10-m walking test; 6MWT: 6-min walking test; TUG: timed-up-and-go test; ∆, change from basal of each outcome variable;

* statistical significance.

We found a high level of correlation between post-block and post-BoNT-A prevalence of clinically significant changes in all functional parameters with a Pearson correlation coefficient (*r*) of 0.98.

Subgroup analysis stratified by EDSS revealed overlap in post-block and post-BoNT-A changes in all variables in patients with EDSS > 3.5, while in subjects with EDSS ≤ 3.5, this predictability emerged for 6MWT and 10mWT only (see Table IV).

**Table IV:** Absolute number of subjects undergoing a clinically relevant change in outcome gait variables stratified according to the Expanded Disability Status Scale (EDSS)

Factor	EDSS > 3.5		*t*-test	EDSS ≤ 3.5		*t*-test
	Post DNB	Post BoNT-A		Post DNB	Post BoNT-A	
10mWT (n)	2	3	0.43	2	2	0.59
6MWT (n)	2	2	0.35	3	4	0.10
TUG (n)	0	0	0.48	1	2	0.00*

Diagnostic nerve block (DNB) predicts the prevalence of positive outcomes after botulinum neurotoxin type-A (BoNT-A) in low-functioning subjects in all variables, while in high-functioning patients it underestimates timed-up-and-go test (TUG) improvement prevalence.

10mWT: 10-m walking test; 6MWT: 6-min walking test;

* statistical significance.

Patients’ distribution of BoNT-A doses, dilution, and formulation is reported in Table V.

**Table V:** Botulinum neurotoxin type-A (BoNT-A) formulation, dilution, and doses used in study population

Subject no.	BoNT-A formulation	Dilution (mL)	Dose (IU)
1	Abo	2.5	250
2	Ona	1	100
3	Abo	2	100
4	Ona	1	75
5	Ona	1	70
6	Ona	1	100
7	Abo	2.5	300
8	Ona	1	100
9	Abo	1.5	200
10	n/a	n/a	n/a
11	Abo	2.5	200
12	Abo	2.5	200
13	Ona	1	70
14	Ona	1	30

Subject 10 did not receive BoNT-A treatment due to unsatisfying diagnostic nerve block results.

Abo: abobotulinumtoxinA; Ona: onabotulinumtoxinA; IU: international units; n/a: not applicable.

## DISCUSSION

The DNB allowed support for the clinical indication to perform BoNT-A treatment on RF muscle in MS patients with unilateral stiff-knee gait. In particular, post-block outcomes provided a forecast of BoNT-A functional impact and a rule-out tool to prevent adverse effects such as excessive strength loss on the tested muscle, which may have had a more impacting role on functional performance than spasticity itself (9).

Based on the results obtained, DNB and BoNT-A lead to an identical improvement in 10mWT and in 6MWT, whereas TUG improvement is more consistent after BoNT-A than DNB. Accordingly, the anaesthetic test may predict BoNT-A treatment outcomes in terms of endurance and walking speed. Interestingly, the TUG improvement after BoNT-A appears higher than after the DNB. In fact, TUG is a more complex functional test, estimating agility, lower limb strength, balance, and fall risk. Therefore, the sudden and short-term alteration of muscle activity and control induced by the DNB may not allow adequate adjustment by the patient to the new neuromotor condition.

Remarkably, a longer-lasting medical history of MS and worse gait performance (more severe EDSS score) correlated with less significant results after DNB on all variables. After BoNT-A, this correlation emerged only for TUG, supporting better efficacy of BoNT-A compared with DNB on endurance and gait speed even in subjects with higher EDSS and longer disease history. These findings may suggest once again a lower expected functional impact of a time-limited test and underline the more significant functional role of BoNT-A. In particular, it is important to note that botulinum toxin is only one component of a multimodal approach to spasticity that relies also on adjuvant post-injection techniques contributing to the overall therapeutic effect. Furthermore, TUG investigates a more complex functional task compared with 6MWT and 10mW, and it is more likely to improve with specific training. On the other hand, gait speed and endurance mainly depend on the “basal” performance status and are more prone to be influenced by maintenance training. This may explain why patients with more preserved function showed higher 6MWT and 10mWT improvement.

In terms of clinical significance of gait modifications, the anaesthetic test managed to predict variations of outcome variables greater than MCID, in the case of 6MWT and 10mWT, or MDC, in the case of TUG, detected after BoNT-A injection. This finding has a crucial clinical value, underling the effectiveness of the nerve block in identifying patients with the expected higher functional improvement, and could be used as an adjunctive criterion to guide integrated and tailored treatment.

Finally, subgroup analysis discloses a meaningful test predictability of clinically significant improvement in patients with lower gait capability (EDSS > 3.5) for all outcome variables. Meanwhile, in the case of higher performance status (EDSS ≤ 3.5), this predictive value concerns 6MWT and 10mWT only. In fact, in this second group, DNB tends to underestimate TUG clinically relevant improvement prevalence subsequent to BoNT-A. Once again, the importance of the rehabilitation treatment (25) and the time needed to settle to a different neuromotor asset after denervation emerges. This aspect is particularly evident for complex functions, as assessed by TUG.

Current literature on nerve blocks in MS spasticity management is scant and not updated, therefore there are no recent and solid data concerning the role of anaesthetic tests prior to BoNT-A treatment in this population (4). However, this technique is more commonly implemented in stroke survivors affected by spastic paresis. In particular, in the specific case of RF spasticity and stiff-knee gait (15), nerve blocks showed a significant level of predictability on all the spatiotemporal gait parameters considered in this study, coherently supporting our results.

Ultimately, it is relevant to note that even in this MS population, similarly to stroke survivors, BoNT-A integrated treatment may allow a transient window of enhanced neuroplasticity (26) to be provided, which could be used to reinforce adaptive learning of more complex functions.

### Limitations

The authors are aware of the limitations of this study. First, the sample size is relatively small, even though compatible with a pilot study, and the observational nature of the work in the absence of a control group may limit the generalizability of the results. Second, the test panel could be implemented with other clinical and neurophysiological assessments to better clarify the role of anaesthetic and BoNT-A denervation. For example, a tridimensional gait analysis could be indicated, allowing measurement of, among other data, the peak knee flexion. Finally, the role of post-injection treatment should be thoroughly investigated, correlating definite interventions with specific outcomes.

In the context of this study, the authors adopted reference values of MCID and MDC calculated in chronic stroke survivors. This choice mainly derives from the lack of MS-specific thresholds in the current literature. Additionally, the pathological pattern of unilateral stiff-knee gait considered in this paper finds its best alignment with hemiplegic patients.

### Conclusions

In cases of uncertain expected benefit from BoNT-A treatment targeting RF, the use of diagnostic nerve blocks could serve as a valuable diagnostic support and rule-out criterion in subjects affected by MS. Moreover, this test may allow the possible clinically relevant improvement after BoNT-A to be forecast with significant predictability.
